# Early Detection of Alzheimer’s Disease: Detecting Asymmetries with a Return Random Walk Link Predictor

**DOI:** 10.3390/e22040465

**Published:** 2020-04-19

**Authors:** Manuel Curado, Francisco Escolano, Miguel A. Lozano, Edwin R. Hancock

**Affiliations:** 1 Polytechnic School, Catholic University of Murcia, 30107 Murcia, Spain; 2 Department of Computer Science and AI, University of Alicante, 03690 Alicante, Spain; sco@dccia.ua.es (F.E.); malozano@dccia.ua.es (M.A.L.); 3 Department of Computer Science, University of York, York YO10 5GH, UK; edwin.hancock@york.ac.uk

**Keywords:** Alzheimer’s disease, neural embedding, random walk, link prediction, brain asymmetries, fMRI networks, directed graphs

## Abstract

Alzheimer’s disease has been extensively studied using undirected graphs to represent the correlations of BOLD signals in different anatomical regions through functional magnetic resonance imaging (fMRI). However, there has been relatively little analysis of this kind of data using directed graphs, which potentially offer the potential to capture asymmetries in the interactions between different anatomical brain regions. The detection of these asymmetries is relevant to detect the disease in an early stage. For this reason, in this paper, we analyze data extracted from fMRI images using the *net4Lap* algorithm to infer a directed graph from the available BOLD signals, and then seek to determine asymmetries between the left and right hemispheres of the brain using a directed version of the Return Random Walk (RRW). Experimental evaluation of this method reveals that it leads to the identification of anatomical brain regions known to be implicated in the early development of Alzheimer’s disease in clinical studies.

## 1. Introduction

Alzheimer’s disease (AD) is a progressive irreversible pathology (neurodegenerative disease) that most frequently affects older people. It has been widely studied by applying network analysis methods to activation patterns using functional magnetic resonance imaging (fMRI). More precisely, the blood oxygen level-dependent (BOLD) signal in fMRI images is a good indicator of activation potentials in different brain regions, and the neuronal activity between the different brain regions can be determined by measuring the correlation between the BOLD activation signals.

These images allow us to study the neuropathophysiology associated with Alzheimer’s disease in its different stages of development. These are usually labelled as: (a) healthy normal control group patients (NORMAL), (b) early mild cognitive impairment (EMCI), (c) late mild cognitive impairment (LMCI) and (d) patients with fully developed Alzheimer’s disease [1]. The pattern of inter-region activation is usually represented as an undirected graph. This network representation of region activity has proved to be a useful tool for understanding the functional working of human brain [2]. Furthermore, in [3], such networks have proved very useful in understanding the neuronal disorders associated with Alzheimer’s disease through the analysis of the intrinsic brain connectivity patterns. Alzheimer’s disease gradually affects the function of different regions of the brain. The symptoms are the progressive impairment of memory, motor tasks, learning and other cognitive abilities. This results in dementia and, finally, death [4]. Converging evidence exists concerning which regions are affected in fMRI images of patients affected by the disease, but it is not totally clear how these cognitive problems or abnormalities are reflected in the functional structure of the whole brain, or how the functional structure changes with different stages of the disease.

In [3], tools from complex network analysis are used with the aim of characterizing the topological structures present in the brain. Specifically, they quantify the functional interaction between all regions of the brain using the mathematical study of activation networks based on graph theory. This methodology offers an attractive approach since it provides useful and effective tools for characterizing network structures together with their intrinsic complexity. There are several approaches that use global and local structures encoded by undirected graphs [4,5,6,7,8,9,10], and machine learning [11,12,13,14].

Graph theory provides meaningful and easily computable measurements to characterize network connectivity and asymmetries associated with both neurological and psychiatric pathologies [4]. In [15], functional brain analysis is performed using features based on the global and local measurement of connectivity.

Almost all the techniques described above use undirected graphs. However, if the origins of the activation patterns are associated with neuronal activity, then directed graphs may be a more natural representation which captures centrality, since they let us measure the temporal causality of activation signals for different anatomical structures in the brain. This implies a new way to establish effective methods for measuring the structural properties representing inter-regional casual networks extracted from fMRI brain data.

To this end in [16], a strategy for the identification of the differences in fMRI activation network structures for patients with EMCI, LMCI and AD is proposed. These directed graphs are combined with entropic measurements to differentiate patients falling into the three affected categories and normal group subjects (healthy patients). They explore three different approaches: (i) a method based on applying linear discriminant analysis for vectors representing the in-degree and out-degree statistics of different anatomical regions, (ii) a method that uses an entropic measure of node assortativity to estimate the asymmetries in the node with in-degree and out-degree, and (iii) a method that selects the most salient anatomical brain regions and uses the degree statistics of the connecting directed links. However, the structure of the directed networks representing the activation patterns and the proposed measures needs to be better developed to understand the differences between healthy subjects and the early development of Alzheimer’s disease when BOLD signal noise is a limiting factor.

Several studies of the early clinical diagnosis of Alzheimer’s disease suggest that there exists asymmetries between right and left brain hemisphere in specific regions of the brain [17,18,19,20], and so there is an imperative to develop methods of analysis that can be used to understand the pattern of asymmetries. In other words, the structure of the directed networks representing the activation patterns needs to be better understood to find the differences between healthy brains and those showing early signs of Alzheimer’s disease when signal noise is present. To reduce the effects of BOLD signal noise and the resulting confusion in the inferred interaction structures between different brain regions, we propose to use a powerful unsupervised network analysis tool, namely the Return Random Walk [21,22]. This method allows us to reduce the inter-class noise while increasing the density of the structure through intra-class link prediction. However, this method uses undirected graphs and it needs a pre-processing step to obtain a better-conditioned input graph (directed network).

To solve this problem, in this paper we propose a method for analyzing asymmetries in different regions of the brain for healthy normal patients (NORMAL) and patients with early mild cognitive impairment (EMCI). In particular, we use the structural properties of directed graphs extracted from fMRI brain data, and using an embedding for inferring an activation graph that is more locally isotropic and harmonic than the original graph (*net4Lap*) [23]. After that, to analyze the brain data in different patients, we demonstrate that the use of a directed version of the Return Random Walk provides a better-conditioned graph whose dense structure allows us to analyze with more precision the asymmetries, expanding it, and propose to quantify this asymmetries through in-out-degree measures of different regions of the brain.

Our experimental results show that the in-degree/out-degree distributions of asymmetries of Normal and EMCI subjects can be useful as a method for early detection of Alzheimer’s disease.

## 2. Materials

### 2.1. Subjects

In this paper, all participants were obtained from the Alzheimer’s Disease Neuroimaging Initiative (ADNI) dataset. We select 147 subjects, where 38 patients were classified as healthy patient (Normal), 47 subjects as EMCI, 32 subjects as LMCI, and 30 as AD. The selected criteria to classify between EMCI and LMCI subjects are described in the ADNI procedure manual (http://www.adni-info.org/).

A subject can present more fMRI acquisitions taken at different time steps. In our study, for each patient we choose only one acquisition (mean). Subjects’ demographic information are summarized in Table 1. This dataset stands at around 1000 patients, but not every patient presents both morphological and functional images. Moreover, the initial status of some subjects in the dataset can be changed (e.g., from Normal to EMCI). For that, those patients were excluded from our study (we only deal with patients that did not change their ADNI classification).

Finally, ADNI initiative dataset has been obtained with a written consent to participate by all participants, according to the recommendations of the Code of Ethics and the Good Clinical Practice guidelines of the World Medical Association (declaration of Helsinki) and and U.S. 21 CFR Part 50 (Protection of Human Subjects), and Part 56 (Institutional Review Boards).

### 2.2. Data Acquisition and Image Preprocessing

In the ADNI study, rs-fMRI data were collected yearly at baseline, one, and two-year follow-ups (three time points in total). The rs-fMRI imaging data scans take advantage of simultaneous multi-slice acceleration for echo-planar images templates with the following parameters: slice thickness = 3.3 mm, matrix = 256 × 256, spatial resolution = 3 × 3 × 3 mm${}^{3}$, number of volumes = 140, and number of slices = 48. From these 140 volumes, the first 10 volumes of each patient were excluded to avoid possible noise related to the balance of the scanner. The remaining volumes were used in the following processing, including slice time correction, motion correction, normalization, and Gaussian spatial smoothing. Finally, to work in a unique reference frame is useful to realign the MRI images with the 64 × 64 fMRI images.

Each image volume is acquired every two seconds with Blood-Oxygenation-Level-Dependent (BOLD) signals. Neuroanatomy experts have been manually drawn several regions of interest (ROIs) from the fMRI voxels. In the dataset have been created 96 ROIs in each fMRI image that correspond to different anatomical regions of the brain and which are labelled with anatomical names to distinguish them (one observation per person, the data is not longitudinal). The correlation between the average time series in different ROIs represents the degree of functional connectivity between regions, and which are driven by neural activities [24].

## 3. Methods and Procedure

**Figure 1:**
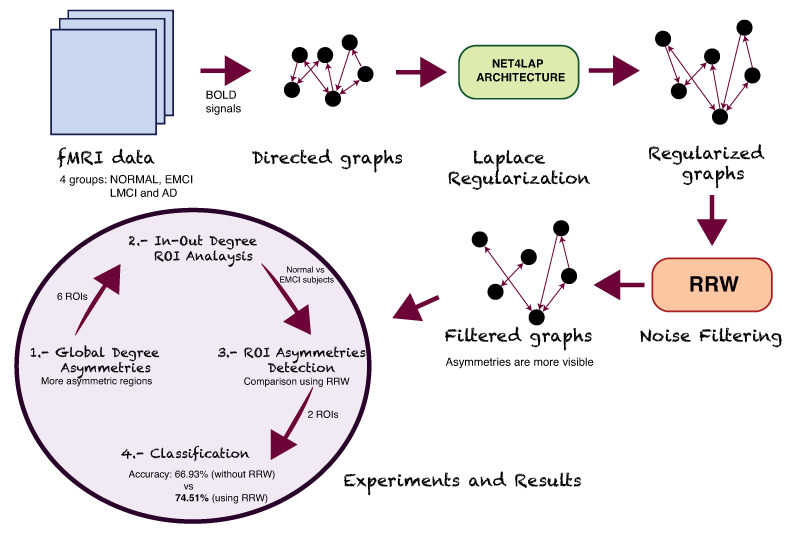
Our proposal: the input data (fMRI data) is transformed to directed graphs and classified in 4 groups (NORMAL, EMCI, LMCI and AD), and then we have applied a graph preconditioning process (Laplace regularization using Net4Lap Architecture and a noise filtering through RRW) to find degree asymmetries in different regions of the brain.

In this paper, we propose to find which regions of the brain are affected in the early stage of Alzheimer’s disease. This is relevant if the early detection of Alzheimer’s disease is attempted. Alzheimer’s is a progressive and slow disease, which takes a long time (20 years) to develop. Recent studies aim to detect and classify the disease with respect to healthy brains using functional magnetic resonance imaging (fMRI). Here we aim to explore the functional relationships between different brain regions using directed graphs. The novel contribution (see Figure 1) is to infer the directed graphs from fMRI information use the net4Lap algorithm (see Section 3.1). The resulting directed graphs have improved local isotropy and harmonic properties when compared to those inferred directly from the thresholded correlation of the BOLD signals for different brain regions. Moreover, the directed graph representation allows us to identify asymmetries between left and right hemispheres of the brain using a directed version of the Return Random Walk. Specifically, we characterize these asymmetries by measuring the in and out-degrees for each region of interest of the brain. Finally, in our experiments (see Section 4) we identify which anatomical brain regions give an early indication of the development Alzheimer’s disease, and we then discuss the meaning of these results (see Section 5) and show them to be consistent with recent clinical studies [17,18,19]. In Section 6 we present the conclusions of our work and discuss future potential directions.

### 3.1. Directed-RRW in fMRI Networks Using *net4Lap*

In this paper, we demonstrate the utility of the Return Random Walk as a tool for early detection of the disease in fMRI activation networks, allowing us to distinguish between Normal subjects and EMCI patients (patients in the early stage of Alzheimer’s Disease or AD). We first compare the differences between the in-degree and out-degree distributions of directed graphs generated using the *net4Lap* algorithm and analyzed using RRW. Then we compare the right and left sections of different regions of the brain (e.g., left vs right temporal cortex), identifying the regions with the strongest asymmetries between the hemispheres. Finally, we use these regions to classify subjects as normal or EMCI using linear discriminate analysis (LDA).

#### 3.1.1. The *net4Lap* Architecture

**Figure 2:**
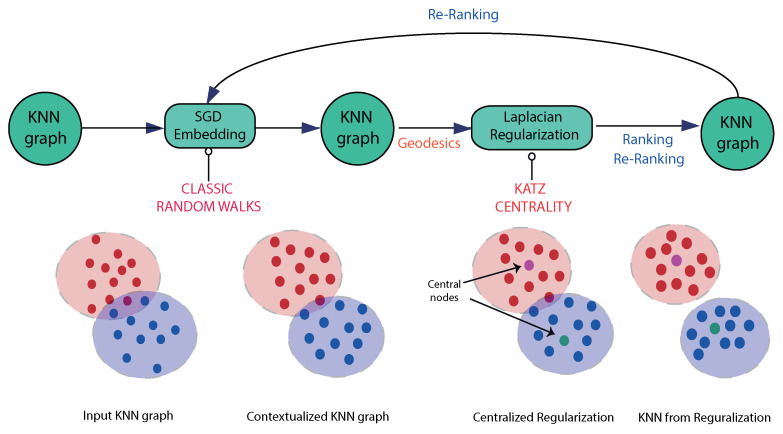
The *net4Lap* Architecture. Given an input *k*NN graph, the process begins with a neural embedding process (stochastic gradient descent with negative sampling) yields a harmonic version that feeds the Laplacian regularizer step. The result is a denser graph suitable either for ranking or for obtaining an improved *k*NN graph which in turns feeds stochastic gradient descend for re-ranking.

To create a directed graph from the fMRI BOLD signal data we use net4Lap. This is a novel architecture for Laplacian-based ranking [23]. This architecture is based on two main ingredients: (i) pre-processing graphs with neural embeddings prior to performing Laplacian ranking, and (ii) introducing a global measure of data centrality (Katz centrality [25]) to modulate a diffusion process on the graph. In this context, ranking can be considered to be an optimization problem where regularization is emphasized. Given an input *k*NN graph G=(V,E), net4Lap (*neural networks for Laplacian-based regularization*), learns an embedding of the nodes of a graph into a vector space via SGD (stochastic gradient descent [26], see Figure 2) from bags-of-paths sampled through different random walks (different empirical studies show that the choice of the algorithm of random walk is not critical provided it has well defined return path policies).

A modified *k*NN graph based on the embedding is more locally isotropic and harmonic than the original one. This modified graph is fed into a Laplacian regularizer based on global centrality. The result is a new *k*NN graph based on node ranking relationships that is re-fed into the stochastic gradient descent neural model for a re-ranking cycle.

As we can see in Figure 2, the architecture of *net4Lap* is the following: (i) a given *k*NN graph (a sparse and potentially structurally noisy graph) is processed by SGD (with negative sampling), (ii) this leads to a neural embedding of the nodes of the graph into a vector space, which and yields a harmonic version of the original graph, (iii) this modified graph is itself fed into Laplacian regularizer. The required neural embeddings are constructed from a bag-of-paths extracted from the original graph using classical random walks. As an output of the initial embedding step, we obtain a denser graph which is locally isotropic and with some structural noise filtered from it. This graph can be used either for ranking or for iteratively obtaining an improved KNN graph [27] which is in turn fed into a further step of stochastic gradient descent for re-ranking.

As a result, we have modified locally isotropic and harmonic graph for use in subsequent processing tasks.

This novel neural-regularization ranking architecture exploits the flexibility and scalability of SGD to pre-process the input graph so that it is well conditioned for Laplacian regularization. In this paper, we investigate the following strategy, namely that the structural noise of fMRI data must be filtered out of the raw activation network because it is easier to compare a diseased brain versus a healthy brain, while it is difficult to compare a brain in an early stage of the Alzheimer disease due to the confusion caused by structural noise. Our approach involves pre-processing a *k*NN graph using neural embedding, where SGD infers a node embedding from a sampling of the graph through random walks with return policies. This gives a modified graph that is both locally isotropic and harmonic (the Laplacian-based ranking minimizes the harmonic loss and absorption probabilities are constrained by Katz centrality [25], which is summarized as follows: a node is important if it is linked to other important nodes). However, this step is only the first part of a pre-processing stage to obtain better graphs for analysis. Later on, we show how to obtain better-conditioned graphs by applying Return Random Walks to remove the structural noise.

**Algorithm.** Given an input kNN graph *G*, net4Lap (Neural Networks for Laplacian-based Regularization) learns an embedding via SGD from bags-of-paths sampled through random walks. A second kNN based on the embedding is more harmonic (locally isotropic) than the original and it feeds a Laplacian regularizer based on global (see Figure 2). This algorithm can be structured in a few steps as follows:

**STEP 1**: Neural embedding and Random Walk. This method is designed through neural embeddings that are built by SGD by sampling bag-of-paths in G=(V,E,W), so that the context from the statistical co-ocurrences with neighboring nodes.

We construct the undirected weighted *k*NN graph *G* as follows: G=(V,E,W), where $W_{ij}=h\left(\frac{1}{\sigma }{\left|\right|x_{i}-x_{j}\left|\right|}^{2}\right)$ are the pairwise affinities between nodes $x_{i},x_{j}\in R^{D}$, h(.) is a sub-Gaussian function, and (i,j)∈E if $W_{ij}>0$. Finally, SGD aims at inferring a function $f:V\to R^{d}$ from:

(1) $$\max_{f}\sum_{i\in V}\log\prod_{j:(i,j)\in E}Pr\left(j|f\left(i\right)\right)$$

where $Pr\left(j|f\left(i\right)\right)=e^{\langle f(i),f(j)\rangle )}/Z_{i}$ (log-probability proportional to correlation) and $Z_{i}=\sum_{k\in V}e^{\langle f(i),f(k)\rangle }$ is the local partition function. Sampling is driven by random walks with some return probability. As result, we obtain a contextualized graph $G^{\prime }=\left(V,E^{\prime },W^{\prime }\right)$, whose weights are locally isotropic, filtering some of the structural noise. We exploit both the flexibility and scalability of SGD to pre-process the input graph.

**STEP 2**: Laplacian Regularization. The similarity relies on the probabilities *A*:

(2) $$A={\left(\alpha \Lambda +L\right)}^{-1}\alpha \Lambda \;,$$

where $L=D^{\prime }-W^{\prime }$ is the Laplacian of $G^{\prime }$, $\Lambda =diag(\lambda_{1},\ldots ,\lambda_{n})$ and α>0 define an affinity function. The harmonic loss predicts that Λ = I is better for moving around dense graphs and Λ = D′ in case of locally sparse graphs.

After that, the problem is planned using a ranking as an optimization problem:

(3) $$\min_{A}Q\left(A\right)={\left|\right|\alpha \Lambda^{1/2}A-\Lambda^{1/2}\left|\right|}^{2}+\gamma trace\left(A^{T}LA\right)\;,$$

where γ = 1. The red term penalizes large deviations associated with linked nodes:

(4) $$trace\left(A^{T}LA\right)=\sum_{i,j}W_{ij}^{\prime }\sum_{k}{\left(a_{ik}-a_{jk}\right)}^{2}\;.$$

The differential absorption flow ($\sum_{k}{\left(a_{ik}-a_{jk}\right)}^{2}$) can only grow when $W_{ij}^{\prime }\approx 0$ or $\left(i,j\right)\notin E^{\prime }$. The local isotropy helps us to constraint this term. The resulting structure contained in $\left\{W_{ij}^{\prime }\right\}$ imposes new links or affinities based on the absorption probabilities.

Setting a balanced Λ we have a clever trade-off that adapts ranking to the underlying manifold, as well as relaxing our optimization problem. The result is a harmonic centralized and regularized graph.

**STEP 3**: Global centrality. The role of global centrality is twofold: (a) capture the underlying density of the manifold using a global measure, and (b) increase the accuracy of re-ranking processes. We build Λ in terms of a more global centrality (Katz):

(5) $$\alpha^{2}\sum_{i}\left[{\left(a_{ii}-1\right)}^{2}+\sum_{k}a_{ik}^{2}\right]C\left(i\right)\;,$$

where *C* is the centrality. Given the red term (Equations (3) and (4)), we must minimize this equation. The result is that global centrality modulates the diffusion process.

In conclusion, we use this method for preprocessing a kNN graph with neural embeddings and SGD infers a node embedding from sampling through RWs, achieving a contextualized graph locally isotropic. The choice of RWs is not critical (if have return probabilities). Laplacian-based ranking minimizes the harmonic loss, and absorption probabilities are constrained by centrality. The Diffusion process is modulated by global centrality (Katz),which is better than Degree centrality. Our approach improves in both ranking and re-ranking (up to 14%). For more mathematical details, see [23].

#### 3.1.2. Directed Return Random Walks

**Figure 3:**
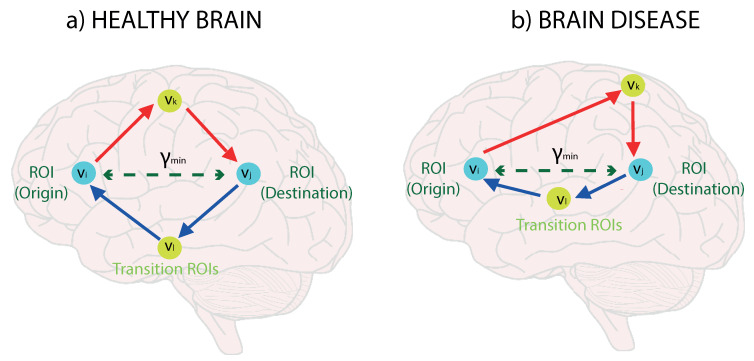
Return random walks (RRW) for detecting ROIs asymmetries. A healthy brain (**a**) shows balanced go and return links (red and blue lines) whereas in an affected brain (**b**) these links have asymmetric weights.

Given the preprocessed graphs from *net4Lap*, we apply a Return Random Walk (RRW) [28]: is a structural filter process, which minimizes the probability of a random walk starting and ending at a given node traverses the inter-class links. As result, we obtain better-conditioned weighted adjacency matrices to identify asymmetries (denser graphs). This method has an easy concept: it works enforcing intra-class edges while penalizing inter-cluster weights, improving the efficiency, and capturing more intra-cluster edges whereas removing inter-cluster edges (noise). The RRW method is designed as follows:

**STEP 1**: Design of We. Given a weighted adjacency matrix *W* we calculate a reweighted adjacency matrix $W_{e}$ as follows: (i) we track the two-step random walks from an origin node $v_{i}$ to a destination node $v_{j}$ via a transition node $v_{k}$, (ii) we return to $v_{i}$ from $v_{j}$, maximizing the probability of returning through a different transition node $v_{l}\neq v_{k}$. For the first step (outgoing from $v_{i}$ to $v_{j}$ through $v_{k}$) we have $p_{v_{k}}\left(v_{j}|v_{i}\right)=\frac{W_{ik}W_{kj}}{d\left(v_{i}\right)d\left(v_{j}\right)}$ as well as a *standard return* $p_{v_{l}}\left(v_{i}|v_{j}\right)=\frac{W_{jl}W_{li}}{d\left(v_{j}\right)d\left(v_{i}\right)}$. This return works well if $v_{i}$ and $v_{j}$ in healthy brains, where left-right paths are balance (see red-blue arrows in Figure 3a). However, the transition node $v_{l}$ for the return could be constrained so that $v_{l}\neq v_{k}$. For this case, travelling out of a region of interest is penalized since the random walker must choose a different path, which in turn is difficult to find on average. Therefore, we obtain $W_{e_{ij}}$ from $W_{ij}$ as follows:

(6) $$W_{e_{ij}}=\max_{k}\max_{\forall l\neq k}\left\{p_{v_{k}}\left(v_{j}|v_{i}\right)p_{v_{l}}\left(v_{i}|v_{j}\right)\right\}\;,$$

i.e., we compute the outgoing and return probabilities for each possible transition node $v_{k}$ (product of independent probabilities) through a different transition node $v_{l}$. We choose the maximum product of probabilities for each $v_{l}$ referred to a given *k* and finally we retain the supremum of these maxima. As a result, when it is selected a far transition region of interest (inter-class) are frequent for a given e=(i,j) (compare asymmetries between red-blue arrows in Figure 3b) its weight $W_{e_{ij}}$ is significantly reduced. The weight $W_{e_{ij}}$ measures the connectedness of two different nodes in a given region or cluster of nodes. If we have a high value, this means that two nodes belong to a region with strong neighbors and thus they are strongly connected. That a region is highly cohesive.

**STEP 2**: Our main hypothesis is that the number of edges is small on average, since the amount of noise (inter-class edges) tends to be small in comparison with the total number of edges. However, patterns can be confused in realistic situations due either to their intrinsic similarity or to the use of an improper similarity measure. This fact leads to a significant decrease of many weights of *W*.

To filter this inter-class noise, we can study the relationship between the sum of different weights of the Return Random Walks and the shortest path (between all pairs of nodes):

(7) $$W_{e_{ij}}^{\prime }=W_{e_{ij}}e^{-(\frac{\gamma_{ij}}{Path_{ij}})}\;,$$

where $Path_{ij}=W_{ik}+W_{kj}+W_{jl}+W_{li}$ and $\gamma_{ij}$ is the shortest path between *i* and *j*.

**STEP 3**: When the Euclidean distance between nodes is high or the path between them is weak (small weight), we have a worse case value of We.

With this equation, we have not attended to the difference between outgoing and return. For this case we take

(8) $$W_{e_{ij}}^{\prime \prime }=\frac{W_{e_{ij}}^{\prime }}{b_{ij}}$$

where $b_{ij}=\frac{W_{ik}+W_{kj}}{W_{jl}+W_{li}}$

Finally, we measure the difference between outgoing and return paths, which gauges the ease with which node *j* is reached from node *i* with a different return path. If the value of *b* is low this will be considered to be an interclass edge because the connectivity is poor.

We expect that the connectedness values to be higher in healthy brains (Figure 3a) because of the better quality of the connections. For instance, connections may be destroyed by Alzheimer’s pathology, and the corresponding probability values are penalized due to the difficulty of the RRW moving through the graph (Figure 3b).

Once we have a filtered the graph, we are going to use it to find asymmetries in our experiments with fMRI data using a measure of imbalance of in and out degree.

#### 3.1.3. In-Out-Degree Measure

Let G(V,E) be a directed graph (a set of vertices connected by edges which have a direction associated with them), where V are all nodes and E are all edges, and the directed edge (u,v)∈E starts at node *u* and ends at node *v*. The directed adjacency matrix *A* is defined as follows:

(9) $$A=\left\{\begin{array}{cc} 1 & \mathrm{if}\;(u,v)\in E\\ 0 & \mathrm{otherwise} \end{array}\right.$$

In this paper, we measure the connectivity of different regions of the brain through the in-degree ($d_{u}^{in}$) and out-degree ($d_{u}^{out}$) of each node *u* corresponding to an anatomical brain region of interest:

(10) $$\begin{array}{cc} d_{u}^{in}=\sum_{u\in V}A_{vu} & \;d_{u}^{out}=\sum_{u\in V}A_{uv} \end{array},$$

where if $A_{uv}=A_{vu}$ implies a bidirectional connection between two regions of the brain, and $A_{uv}\neq A_{vu}$ implies a unidirectional connection.

To measure the asymmetries between two regions (left *l* and right *r*) of the same anatomical area of the brain, we define the difference or ratio *in-out-degree* as follows

(11) $$\begin{array}{cc} \mathrm{In}-\mathrm{Out}-{\mathrm{Degree}}_{l}=d_{l}^{in}-d_{l}^{out} & \;\mathrm{In}-\mathrm{Out}-{\mathrm{Degree}}_{r}=d_{r}^{in}-d_{r}^{out} \end{array},$$

and which quantity the degree dissimilarity or asymmetry of two subregions of the same area of the brain.

## 4. Experiments

In this section, we describe our method for early Alzheimer’s detection in fMRI activation networks for Normal and EMCI subjects. We first compare the difference between in-Degree and out-Degree of the directed graphs generated with *net4Lap* and the RRW (The code is available in: https://github.com/manuelcurado/ALZcode). Then we compare right and left portions of different regions of the brain, identifying those regions with the strongest asymmetries between both left and right hemispheres. Finally, we evaluate whether these anatomical regions can be used to classify subjects as normal or EMCI using linear discriminant analysis (LDA).

### 4.1. Synthetic Experiments

**Figure 4:**
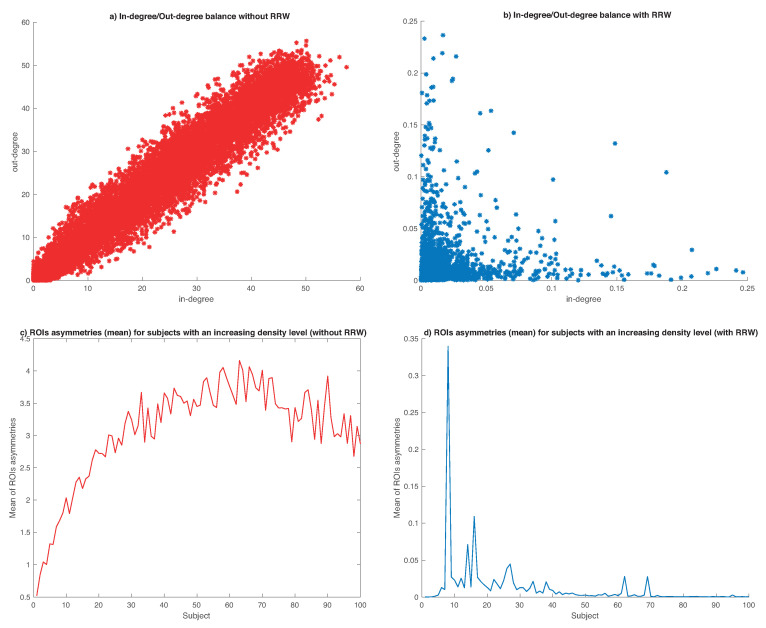
Synthetic experiments.Top (**a**,**b**): Comparison of 500 subjects without RRW (in red) and using the algorithm (in blue). Bottom (**c**,**d**): Evolution of asymmetries with an increasing density without RRW (in red) and using RRW (in blue).

To evaluate the implications of our algorithm, we generate a synthetic dataset of 500 subjects (or graphs) with their respective 96 regions of interest or areas of the brain (nodes) with different levels of edge density (links between different regions of interest) whose values are between 0 and 1 randomly. This dataset does not distinguish between different groups (Normal, EMCI, LMCI or AD). In other words, all subjects are mixed. To understand the difference between a diseased or healthy brain, the main characteristic of this disease is a progressive disconnection of the areas of the brain, represented through sparser graphs (links are missing, or its weights are weak). In contrast, a dense graph represents a healthy brain with strong links. From this point, in Figure 4a,b we can see the impact of using RRW in our dataset. If we do not use RRW (Figure 4a), the in-degree/out-degree distribution is symmetric (balanced in-degree/out-degree distribution, in the diagonal), being very difficult to differentiate between dense and sparse graphs. In contrast, using RRW (in Figure 4b) we can see the expanding effect of the algorithm, where ROIs are more asymmetric. This situation helps us to differentiate better between normal and affected patients.

As complementary experiments, we select a random subset of 100 subjects and we represent (in density ascending order) their sum of degree differences (in-degree and out-degree) of all ROIs of each subject. A high value of this sum means a more asymmetric distribution (affected brain). In Figure 4c,d, we can see how the asymmetries are higher with denser graphs (in Figure 4c), but after applying RRW (in Figure 4d) we increment the asymmetry of dense graphs (sparse graphs), and increase the possibility of detecting and classify between healthy and affected patients.

**Figure 5:**
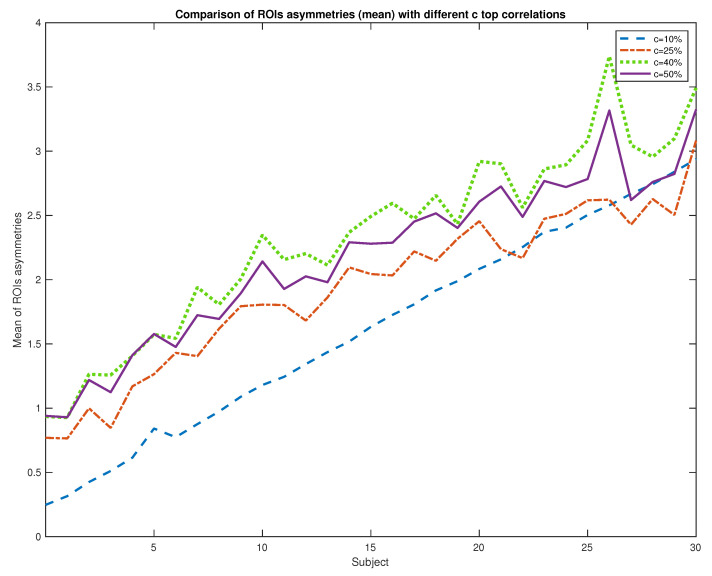
Synthetic experiments. Comparison of different levels of correlations.

In the following subsection, we can see the difficulty of differentiating between healthy patients and the early stages of the Alzheimer disease due to the disconnection of links of the graph structure (sparse graphs). For a correct study, we retain the highest c correlation coefficients. For that, we select a subset with the 30 sparser subjects, and we compare the evolution of asymmetries (in decreasing sparse order) with different coefficients values (c=10,25,40 and 50%) in Figure 5. We choose the threshold c=40% (maximize the asymmetries to make easier subsequent tasks as classification).

### 4.2. Real Data: Creating Meaningful Directed Graphs from fMRI Data

In this subsection, we prove our hypothesis with real data. We use the fMRI data coming from the ADNI initiative [29].

We construct a directed graph with 96 nodes using the *net4Lap* method, where we deal with the sign of the time-lag between different time series for different ROIs and the magnitude of the correlation. Moreover, to model causal interaction, we use the time lagged cross-correlation values for the average time-series for all pairs of regions of interest in the directed graph. For that, we use a kNN graph, varying *k* from 5 to 50. Then, we applied a directed version of Return Random Walk to measure the connectivity between two regions: a high value means both nodes have a good degree of connection and probably belong to the same cluster or they share an important workflow (information). We use *k* = 15, since this value is more adequate in similar graphs problems using Return Random Walk [28,30].

With directed-RRW graphs, we focus on his adjacency matrix, where the diagonal elements are set to zero, and then apply a threshold to retain only the maximum values of edges with the top c=40% of correlation coefficients (as we have seen on synthetic experiments), to obtain a binary directed adjacency matrix with the most relevant ROI information for each subject. In addition, if a ROI have missing time series data is discarded. The resulting dataset has four categories which represent different levels of the disease. In this paper, we are interested in patients in an early Alzheimer’s stage. For that, our experiments deal with 47 EMCI and 38 normal healthy control subjects.

### 4.3. Global Degree Asymmetries

**Figure 6:**
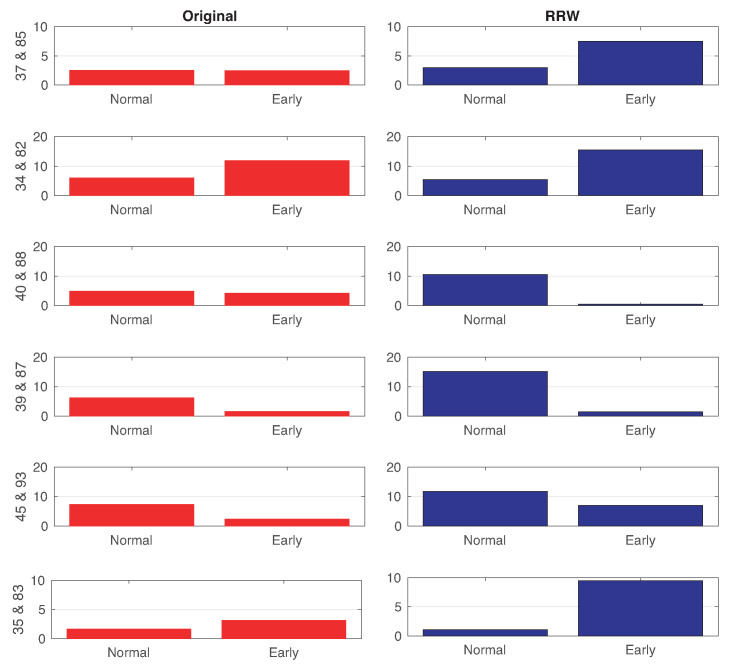
Differences between the averages of Normal and EMCI subjects in the original directed graph after *net4Lap* (in red) versus directed Return Random Walk graph (in blue). Rows are the area of brain represented by the difference between the two different ROIs. The bars represent the difference between two regions of the same anatomical region of the brain, and left text labels are these number of regions (see Table 2) implied in each case.

In the first experiment, we analyze the degrees of all ROIs and compare the average differences between left and right hemispheres of the same regions of the brain. The problematic interpretation of the rank order of centrality if nodes do not differ much in centrality, resulting in a rank order mostly random is partially solved with our method through the asymmetries expanding (the centrality is very significative when we retain the top 40% correlations). First, we calculate the degrees (in and out) of all graphs (or patients) from the normal and EMCI subjects. Then we sort the differences between in-degree and out-degree values in ascending order and later we rank the nodes by assigning a value from 96 to 1 to each existing ROIs, obtaining significant differences in the rank order. Finally, we compare the difference between left and right ROIs of the same area of the brain. In Figure 6, we show the six ROIs or regions with the highest difference between the averages for the normal and EMCI subjects. These results are obtained using the directed Return Random Walk of a *k*NN graph with respect to the originally directed graphs after just applying *net4Lap’s* neural embedding but without computing RRWs. We can see relevant asymmetries in the RRW in comparison with the original graphs. The information for these regions is given in Table 2. In the table, the Brodmann Area refers to the Brodmann’s code associated with each of our ROIs.

### 4.4. In-Degree/Out-Degree ROI Analysis

**Figure 7:**
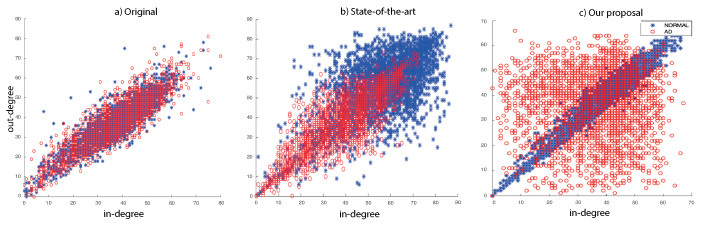
The in-degree/out-degree distribution of all nodes in the original data (**a**), state-of-the-art (**b**) and without directed RRW graphs (**c**). We compare healthy normal subjects (in blue) with AD patients (in red).

In the second experiment, we explore the in-degree/out-degree scatter for our dataset. In the state-of-the-art works, it is possible to obtain improved classification accuracy comparing (in directed or undirected approaches) Normal versus AD patients (see Figure 7b) with respect to the original data (see Figure 7a). Our method finds a better expansion of the asymmetries in in-out degree distributions, as we show in Figure 7c.

**Figure 8:**
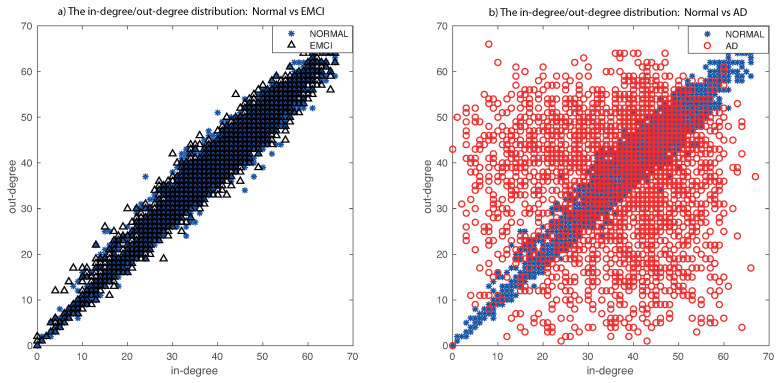
The in-degree/out-degree distribution of all edges in directed RRW graphs. In (**a**), we compare healthy normal subjects (in blue) with EMCI patients (in black) and, in (**b**), we compare healthy normal subjects (in blue) with AD patients (in red).

The main problems with the state-of-the-art methods are that they focus on two different approaches: (i) the comparison between early (EMCI) and late (LMCI) patients, obviating the interesting comparison between normal subjects and patients in an early stage of the disease [5,31], and (ii) the comparison is centered on subjects between normal and mild cognitive impairment (MCI, a join set of EMCI and LMCI), losing the emphasis on early detection [32,33,34,35,36]. The comparison between EMCI and normal subjects is difficult due to the almost complete overlap of the degree distributions. We compare normal vs EMCI (in Figure 8a) and normal vs Alzheimer’s disease (in Figure 8b) after applying our method. This figure (and previous one) shows the relationship between in-degree and out-degree links. In AD patients, there are areas of the brain where one of these degrees is bigger with respect to another degree. These asymmetries are reflected through an expanded distribution (in red). In the previous figure, we can compare the difference between healthy and AD patients of the original data, our method, and the state of the art (our method achieves a more expanded distribution). However, in this Figure 8, we show the main problem: to differentiate between Normal and patients in an early stage of the disease. Our method increases the scatter of the EMCI about the diagonal in comparison with the state-of-the-art methods, but it is still very difficult to compare distributions. Our hypothesis is that there exist more asymmetries in specific anatomical regions of the brain. For this reason, we perform a deeper analysis of the ROIs involved or implicated in the early phase of Alzheimer’s disease in the following experiments.

### 4.5. ROI Asymmetries Detection

**Figure 9:**
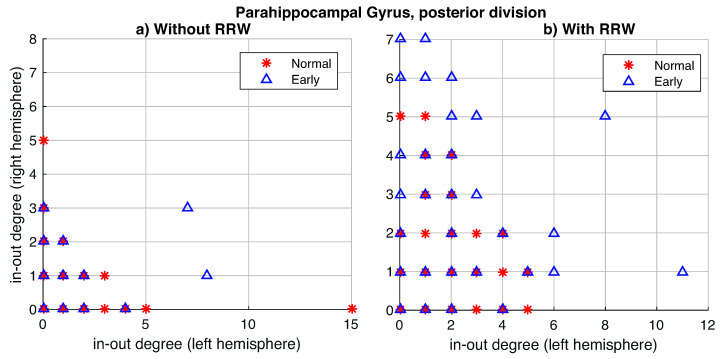
The in-degree/out-degree distribution of the *Parahippocampus Gyrus, posterior division*. Comparison without RRW (**a**) and using directed RRW (**b**). We compare healthy normal subjects (in red) with Early or EMCI patients (in blue).

**Figure 10:**
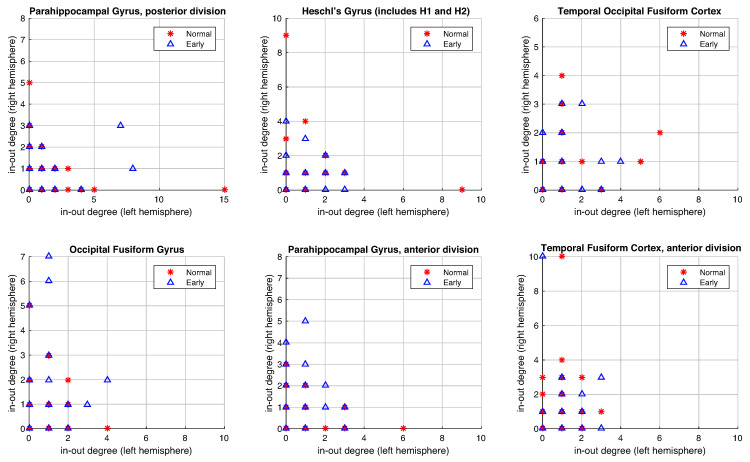
The in-degree/out-degree distribution of top 6 asymmetric regions of connectivity in the original graph (without directed RRW graphs). We compare healthy normal subjects (in red) with EMCI subjects (in blue).

**Figure 11:**
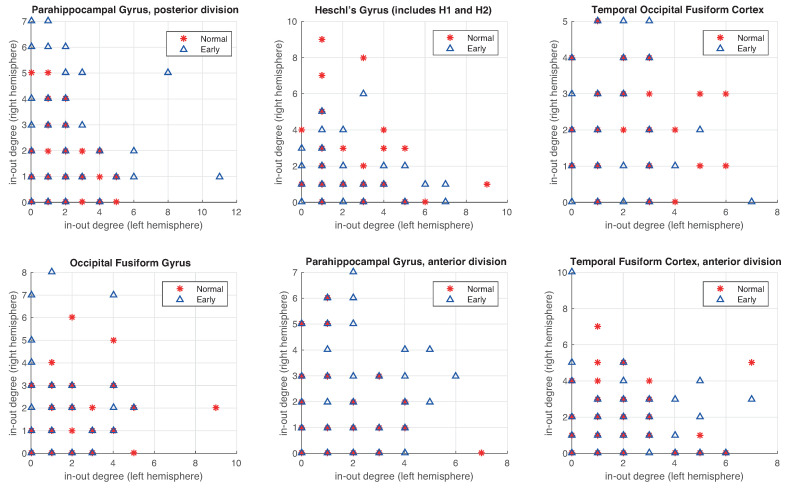
The in-degree/out-degree distribution of the 6 most asymmetric regions of connectivity using directed RRW graphs. We compare healthy normal subjects (in red) with EMCI subjects (in blue).

In the third experiment, we analyze the in-degree/out-degree distribution for regions of the brain with a high level of asymmetry (in Table 2). The representation of the symmetric left-right hemisphere distribution would be a distribution where all points are close to the diagonal or in the origin point (0,0). For that, farther the point concerning this symmetry balance, the bigger the asymmetry of this value. First, we show the importance of using the RRW to emphasize or amplify the evidence for the existing asymmetries in the graphs, for instance, in a specific area (Parahippocampal Gyrus posterior division), where the distribution between both left and right hemispheres in-out-degrees are sparser (Figure 9a) than without RRW (Figure 9b). In other words, we compare the in-degree/out-degree distribution of the six anatomical brain regions with the largest difference between left and right hemisphere obtained in the previous experiments. In Figure 10 we show the results for the original graph (without directed RRW). In all cases, in-out-degree differences are close to zero with respect to both hemispheres. However, in Figure 11, the distribution is sparser and patients with early symptoms have more asymmetries (far from the diagonal). In particular, two regions namely (a) Parahippocampal Gyrus and (b) anterior and posterior division show this effect strongly.

These results are very consistent with previous studies [17,18,19]. Moreover, the regions studied are included in the Entorhinal cortex. This region is not considered to be the first area of the brain to be affected in the development of Alzheimer’s disease [37,38], but the disease leads to asymmetries in the volume of the Entorhinal cortex.

**Figure 12:**
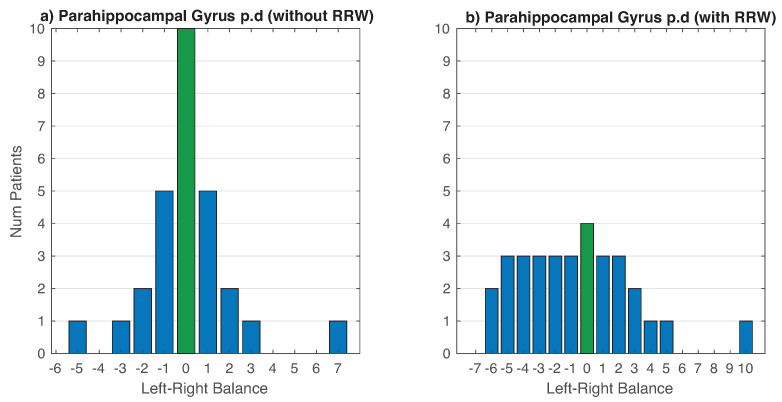
Comparison: the use of RRW in the balance of symmetries in both hemispheres of the brain. In (**a**), we do not use RRW and in (**b**), using RRW. Green bar is the center (balance) between hemispheres.

Finally, we show in Figure 12a the effect of using RRW in the balance between hemispheres. In Figure 12 left we can see how almost patients are concentrated in the point of balance (green bar). In contrast, if we applied RRW (Figure 12b), there is a sparser distribution (expanded asymmetries).

### 4.6. Alzheimer’s Classification

To conclude, we perform a linear discriminant analysis (LDA) on the two classes of our dataset (normal and EMCI subjects) as in [16] for a better comparison with the state-of-the-art. In this work, we represent the graph with a multidimensional feature vector that includes the in-degree/out-degree of left and right ROIs of the six most asymmetric areas as a measure of asymmetric level (the representation of the difference between them). We analyze the classification accuracy obtained using LDA, both with and without the directed RRW. In this classification experiment, we use both normal and EMCI subjects for training. The performance of LDA classifier is computed by using 10-fold cross-validation. In other words, we randomly divide our 85 subjects (38 normal and 47 EMCI) into 10 disjoint subsets of the same size. Then, we remove a subset and we train LDA using the remaining subsets. The selected subset is retained and used to measure the classification accuracy of the trained classifier. This method is repeated 10 times (10-CV), removing different subsets in each trial, and taking the average of all the classification results. Finally, we obtain an accuracy of 74.51% with directed RRWs versus accuracy of 66.93% without them (Table 3). Other studies that use directed graphs (as [16]) obtain good results (90% of accuracy), but their comparison is between AD and Normal patients.

## 5. Discussion

There is an extensive literature about how to study Alzheimer’s disease from a computer science perspective. The main goal is to obtain good discrimination between different subjects and thus a good classification accuracy between healthy subjects and subjects with different stages of the Alzheimer pathology. Our study makes two distinct contributions:

- We characterize the BOLD signal data using directed graphs. We consider this to be a more natural representation for brain structure. Moreover, graph theory provides meaningful and easily computable measurements to detect connectivity abnormalities, in contrast with other state-of-the-art methods [4,5,6,7,8,9,10].
- The most difference between our methodology with respect to the state of the art is the comparison. Our goal is to compare normal subjects with those suffering from early mild cognitive impairment (Normal vs. EMCI). The most common strategy in the literature is to mix early and late phase (EMCI+LMCI cognitive impairment data) into a unique group (MCI) [39,40,41,42,43]. This is because the objective of this work is the early detection of the disease and determining which regions of the brain are involved in this phase. This represents a novel direction with respect to the state-of-the-art.

We have proposed a method for refining the directed graphs extracted from fMRI brain data using net4Lap embedding. This yields more locally isotropic and harmonic graphs. These subjected to further analysis using a directed version of the RRW, which amplifies the asymmetries between the left and right hemispheres of some affected regions of the brain. We have evaluated the importance of our method in Section 4.5, and show that the classification accuracy improves by 8% (see Section 4.6).

Finally, we have highlighted two specific regions (Parahippocampal Gyrus—anterior and posterior division) as strong indicators of early AD (this is consistent with some clinical studies [17,18,19,20]). In [16], these regions are included in the list of the ten anatomical regions with the largest entropy differences between normal and AD subjects. Related work [31,32,33,34,35,36,44] has highlighted the asymmetries between right and left hemispheres of the hippocampus as one of the regions with more relevance (beside hippocampal subfields and entorhinal cortices), but they use a subset of MCI patients.

**Limitations:** Our current study is limited by two factors. Besides MRI and PET, there are also other modalities of data as APOE. However, we decide to discard this modality because not every subject has information of all modalities and the number of patients with all modalities available is too small for reasonable classification. The second limitation is the lack of methods that separate MCI groups (EMCI and LMCI) with directed graphs in their experiments (see [14,40,45]). Moreover, other limitations include the cross-sectional nature of this database and the absence of longitudinal RS-fMRI data.

## 6. Conclusions

In conclusion, in this paper we propose a novel approach to the analysis of fMRI regional brain interaction networks with the aim of detecting the Alzheimer disease at an early stage. In contrast with the state-of-the-art, which they focus on the classification of full Alzheimer’s versus healthy normal patients, or the aggregate of subsets LMCI and EMCI patients in MCI patients, we focus on distinguishing normal and EMCI patients. We follow a strategy based on directed graphs because this representation is more natural for brain structure. Our method is based on a pre-processing step where the *net4Lap* embedding is applied to the input directed graph to obtain refined graphs, which are more locally isotropic and harmonic than the input ones. However, we still face an important obstacle due to the overlap between the distributions of normal and EMCI subjects. To reduce this problem, we use a directed version of the RRW to filter inter-class noise, and to highlight the regions with significant asymmetries. In other words, this method helps us to discriminate between brain regions or areas which are implicated in the early stages of Alzheimer’s by emphasizing or amplifying the existing asymmetries in the graph.

In our experiments, we have detected asymmetries in the degree distributions between the left and right regions of the brain associated with the same anatomical area. We have isolated six potential brain regions, and highlight two specific regions with an important asymmetry (Parahippocampal Gyrus, anterior and posterior division), which are indicators of the early development of Alzheimer’s and are consistent with clinical studies. Finally, we classify the data, distinguishing between EMCI and normal subjects. Our approach increases the classification accuracy from 66.93% to 74.51% (8%). This is a significant improvement that demonstrates the usefulness of using the RRW in activation structure analysis problems. In future work, we will study how these asymmetries evolve to late mild cognitive impairment and develop tests for possible changes in different anatomical regions associated with the development of Alzheimer’s disease.

## Figures and Tables

**Table 1:** Demographics and neuropsychological data for all groups of patients (in Gender, M is Male and F is Female, and SD is Standard Deviation).

	Number of Patients	Gender	Age Range (Years)	Mean Age (SD)
Normal	38	17 M/21 F	66–87	74.19 (±5.8)
EMCI	47	19 M/28 F	62–88	71.59 (±7.4)
LMCI	32	18 M/14 F	61–85	72.42 (±6.9)
AD	30	11 M/19 F	61–90	71.56 (±7.5)

**Table 2:** ROIs with six highest asymmetries left-right hemispheres. Last columns are the p-value of pairwise comparisons measured by Mann-Whitney U test (see Figure 6, the original dataset without RRW vs dataset after applying RRW, where low *p*-values indicate large difference in the average of the two groups.).

Area of Brain	#ROI (Left)	#ROI (Right)	#Brodmann Area	*p*-Value (Original)	*p*-Value (RRW)
Temporal Fusiform Cortex, anterior division	37	85	36	0.069	<0.001
Parahippocampal Gyrus, anterior division	34	82	36	0.089	0.009
Occipital Fusiform Gyrus	40	88	19	0.071	<0.001
Temporal Occipital Fusiform Cortex	39	87	37	0.077	0.008
Heschl’s Gyrus (includes H1 and H2)	45	93	48	0.058	0.007
Parahippocampal Gyrus, posterior division	35	83	30	0.121	<0.001

**Table 3:** The LDA 10-cross validation classification accuracy with information extracted from the 6 ROIs with the highest degree of asymmetry between left and right hemispheres for normal and EMCI subjects. Our best result using RRW is 74.51% (in bold).

LDA	Accuracy	Sensitivity	Specificity	Precision
Non-filtered	66.93% (±0.54)	49.36%	80.97%	68.12%
With RRW	**74.51% (±0.81)**	66.82%	80.42%	74.11%
